# Dissection of P2X4 and P2X7 Receptor Current Components in BV-2 Microglia

**DOI:** 10.3390/ijms21228489

**Published:** 2020-11-11

**Authors:** Mira Trang, Günther Schmalzing, Christa E. Müller, Fritz Markwardt

**Affiliations:** 1 Julius-Bernstein-Institute for Physiology, Martin-Luther-University, D-06097 Halle-Wittenberg, Germany; mira.trang@web.de; 2 Institute of Clinical Pharmacology, RWTH Aachen University, D-52074 Aachen, Germany; gschmalzing@ukaachen.de; 3 Pharmaceutical Institute, Pharmaceutical & Mediinal Chemistry, University of Bonn, D-53121 Bonn, Germany; christa.mueller@uni-bonn.de

**Keywords:** P2 purinergic receptor, P2X7 receptor, P2X4 receptor, voltage clamp, microglia

## Abstract

Microglia cells represent the immune system of the central nervous system. They become activated by ATP released from damaged and inflamed tissue via purinergic receptors. Ionotropic purinergic P2X4 and P2X7 receptors have been shown to be involved in neurological inflammation and pain sensation. Whether the two receptors assemble exclusively as homotrimers or also as heterotrimers is still a matter of debate. We investigated the expression of P2X receptors in BV-2 microglia cells applying the whole-cell voltage-clamp technique. We dissected P2X4 and P2X7 receptor-mediated current components by using specific P2X4 and P2X7 receptor blockers and by their characteristic current kinetics. We found that P2X4 and P2X7 receptors are activated independently from each other, indicating that P2X4/P2X7 heteromers are not of functional significance in these cells. The pro-inflammatory mediators lipopolysaccharide and interferon γ, if applied in combination, upregulated P2X4, but not P2X7 receptor-dependent current components also arguing against phenotypically relevant heteromerization of P2X4 and P2X7 receptor subunits.

## 1. Introduction

Microglia cells as resident macrophages constitute the immune system of the brain and spinal cord. They are activated under pathological conditions such as ischemia, trauma and neurodegeneration [1,2,3,4]. Activated microglia cells are then involved in inflammation, pain sensation and tissue remodeling [5,6]. Microglia cells are activated by binding of pathogen-associated molecular patterns (PAMPs) and danger-associated molecular patterns (DAMPs) [3]. The DAMPs include ATP which is released from damaged or inflamed tissue [7]. Microglia cells not only are activated by ATP, but also secrete ATP themselves by mechanisms that have not yet been fully elucidated [4,8,9]. Receptors for extracellular ATP are the G-protein coupled P2Y and the ionotropic P2X purinergic receptors [10,11]. The P2X receptor family consists of seven subtypes [12], of which the P2X4 receptor (P2X4R) and the P2X7 receptor (P2X7R) are involved in microglia activation [2,4,13,14]. Under pathological conditions, the expression of the P2X4R [14,15,16,17,18,19] and the P2X7R [20,21,22,23,24] is increased. Stimulation of P2X7 and/or P2X4 receptors leads to chemotaxis of microglia towards regions of cell damage [25,26], microglia-mediated cytokine release [22,27,28], neuropathic pain [29,30], and phagocytosis of debris [31,32]. Inhibition of P2X4 [14,18,30,33,34] or P2X7 receptors [24,27,29,35] is considered a possible therapeutic principle to limit neuronal damage and pain. However, blocking these P2X receptors may also hamper beneficial effects of P2X4/7-dependent microglia activation [23,32,36].

The coexpression of P2X4 and P2X7 receptors in microglia raises the question whether P2X4 and P2X7 receptors are activated in parallel, i.e., independently from each other or if there is a physical and/or functional interaction of the two P2X receptor subtypes, as has been convincingly shown for other P2X receptor subtype combinations [37]. This may be of therapeutic relevance if P2X4 and/or P2X7 receptors are modulated with drugs to alleviate microglia-dependent pathological processes in the central nervous system [14,18,24,29,30,32,34,35]. There are conflicting reports regarding the interaction between P2X4 and P2X7 subunits in different cell types [37,38,39,40,41,42].

Here, we dissected ATP-induced P2X4- and P2X7-dependent ion current components based on agonist- and antagonist sensitivity, ion current kinetics, and modification of their expression by lipopolysaccharide (LPS) and cytokines to reveal a possible interaction of both P2X receptor subtypes.

## 2. Results

### 2.1. ATP-Dependency of Current Amplitudes

**Figure 1:**
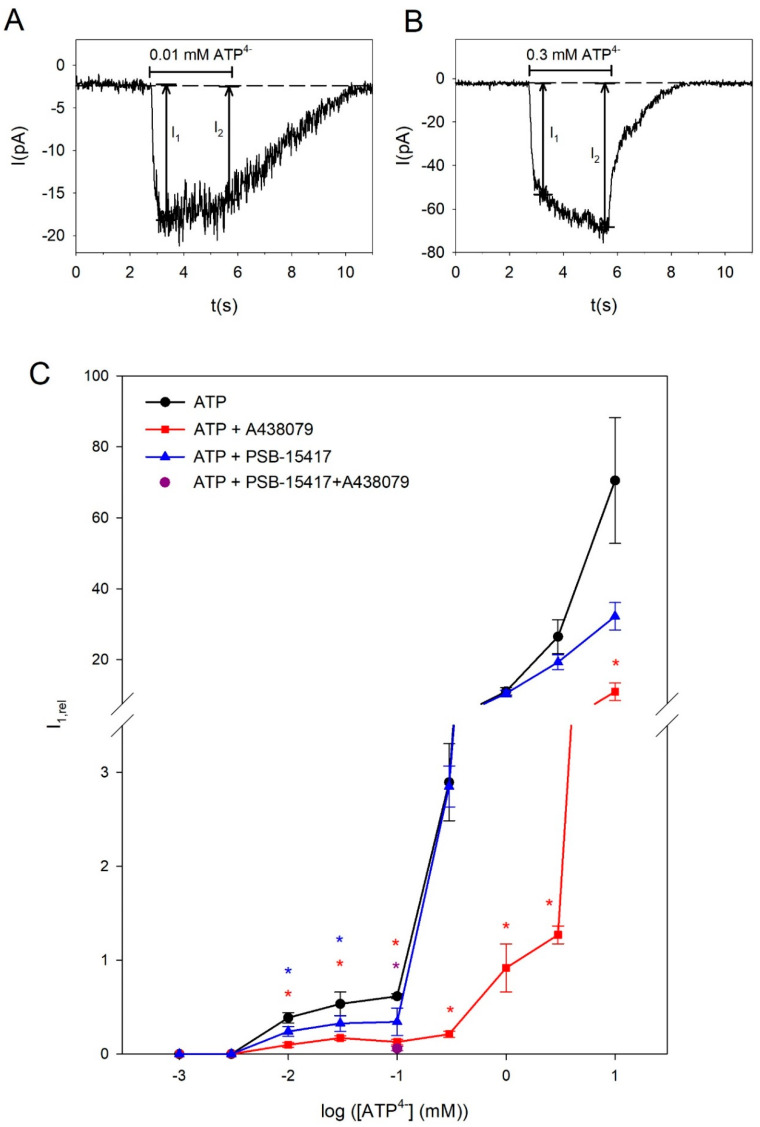
Concentration-dependence of ATP-induced currents in BV-2 microglia cells. (**A**,**B**) Representative current traces elicited by applying 0.01 mM or 0.3 mM ATP^4−^ as indicated. The determination of I_1_ and I_2_ is illustrated. (**C**) The curves show the ATP^4−^ concentration dependence in the absence and presence of 10 µM of the P2X4R blocker PSB-15417 or 10 µM of the P2X7R blocker A438079 as indicated. Current amplitudes I_1_ were normalized to a preceding application of 0.1 mM ATP^4−^. Data are means ± SEM of 6–231 cells. Significant differences from the ATP^4−^ alone curve are marked by asterisks (blue: PSB-15417, red: A438079).

Since the P2X4R- and P2X7R-dependent currents show clear dependencies on the ATP concentration, we measured ATP-induced whole cell currents by applying ATP for 3 s at a holding potential of −40 mV. We related the concentration dependencies to the free ATP^4−^ concentration ([ATP^4−^]), since the agonist at both receptors is ATP^4−^ rather than CaATP^2−^ [43,44]. At low ATP^4−^ concentrations, such as 0.01 mM, after a rapid activation within <400 ms, slowly desensitizing current components were measured (Figure 1A). In contrast, at higher ATP^4−^ concentrations, such as 0.3 mM, the currents exhibit a slow increasing currents component (Figure 1B). The amplitude I_1_ of the fast activating current component was quantified as peak current amplitude for the desensitizing currents and 400 ms after ATP application for the non-desensitizing currents (Figure 1A,B).

Figure 1C shows the biphasic dependency of the ATP^4−^ concentration on the amplitude of the fast activating current component I_1_. We assume that the small currents activated at low ATP concentrations <0.3 mM ATP^4−^ are carried by P2X4 receptors and the currents evoked by [ATP^4−^] ≥ 0.3 mM are mainly due to P2X7R activation. To verify this, we measured [ATP^4−^] concentration dependencies of the whole cell currents with additional application of the selective P2X4R blocker PSB-15417 [42] or the selective P2X7R antagonist A438079 [45]. Indeed, the currents evoked at low [ATP^4−^] were significantly attenuated by PSB-15417. A438079 inhibited the currents in the concentration range from 0.01 to 10 mM ATP^4−^, indicating that currents elicited by low [ATP^4−^] might also be partially mediated by P2X7. Application of 0.1 mM ATP^4−^ together with PSB-15417 and A438079 reduced the relative current I_1_ to 5.9 ± 2.2% compared to the foregoing ATP^4−^ application without the blockers (Figure 1C). This demonstrates a nearly full block of the current by the P2X4R and P2X7R blockers and indicates that other P2X receptors hardly contribute to the ATP-dependent currents in BV-2 cells.

### 2.2. ATP-Dependency of Current Kinetics

Another criterion for distinguishing between P2X4R and P2X7R current components is the different current kinetics. To quantify the different kinetics of the slow current components, a relative late current was calculated as the difference in current after 3-s ATP application and I_1_, related to I_1_ (Equation (1)):

I_late,rel_ = (I_2_ − I_1_)/I_1_ (1)

where positive values indicate desensitizing and negative values slowly activating currents.

**Figure 2:**
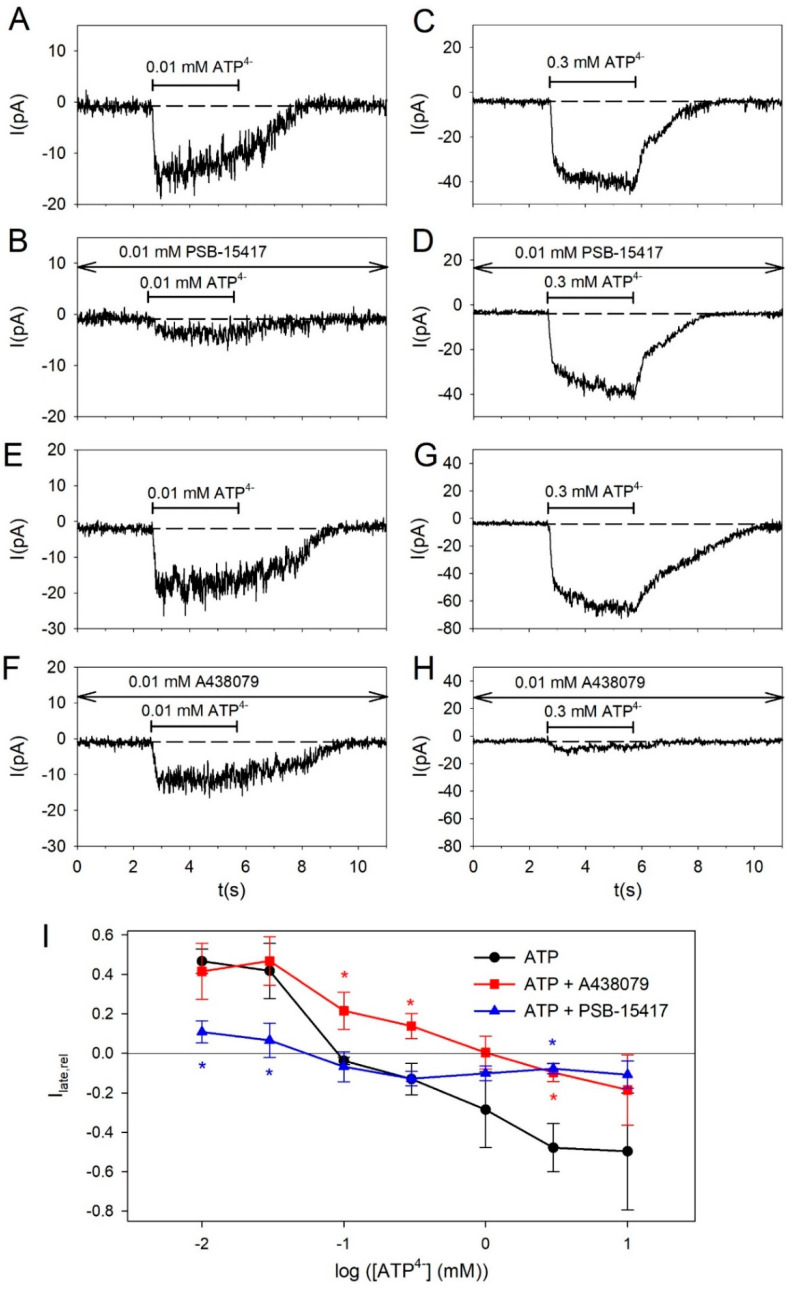
Concentration-dependence of kinetics of ATP-induced currents. (**A**–**H**) Representative current traces elicited by 0.01 or 0.3 mM ATP^4−^ without and with the P2X4R blocker PSB-15417 and the P2X7R blocker A438079 as indicated. (**I**) Relative current amplitudes of the late current I_late,rel_ were calculated according to Equation (1). The curves show the concentration dependence on ATP^4−^ in the absence and presence of 10 µM PSB-15417 or 10 µM A438079 as indicated. Means ± SEM of 6–227 cells. Significant differences from the ATP^4-^ alone curve are marked by asterisks (blue: PSB-15417, red: A438079).

The concentration dependency of this current component is shown in Figure 2. A438079 shifts I_late,rel_ to more positive values for [ATP^4−^] > 0.03 mM, indicating a blockage of the slowly activating P2X7R-mediated current. PSB-15417 decreased I_late,rel_ at low [ATP^4−^] < 0.1 mM pointing to a block of the desensitizing P2X4R-mediated current component. However, at high ATP concentrations, PSB-15417 also blocks the slowly activating current component that is supposed to be mediated mainly by P2X7 receptors.

**Figure 3:**
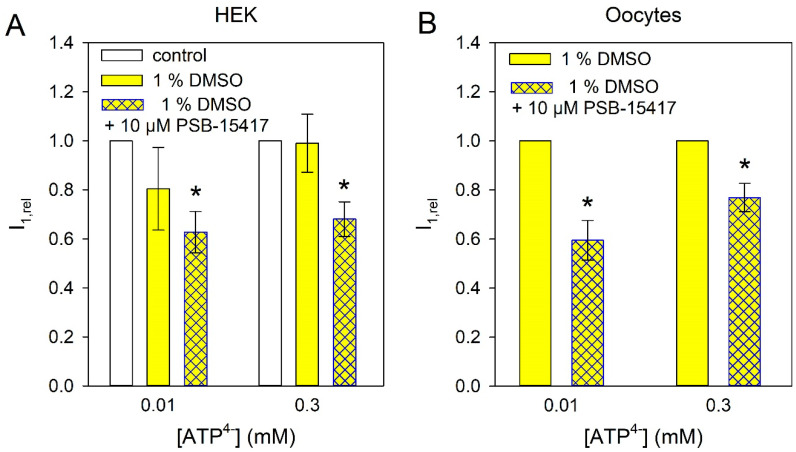
Effect of PSB-15417 on P2X7R-dependent ion currents. Currents were measured in HEK cells expressing hP2X7 (**A**) and in Xenopus oocytes expressing mP2X7 (**B**). Means ± SEM of 5–12 cells. Significant differences from the control are marked by asterisks.

To clarify whether this effect is due to a functional P2X4/P2X7 heteromer that contributes to the current at high [ATP^4−^] or to a blocking effect of PSB-15417 on P2X7, we measured the effect of PSB-15417 on cells expressing P2X7R alone. Both in HEK293 cells expressing the hP2X7R and in oocytes expressing the mP2X7R, 10 µM PSB-15417 (which exhibits an IC_50_ of <0.1 µM at the mouse P2X4-dependent Ca^2+^ influx, [46]) inhibited P2X7R-dependent currents by about 25 to 40% (Figure 3), which may explain the effect of PSB-15417 on BV-2 cells at high ATP concentrations.

**Figure 4:**
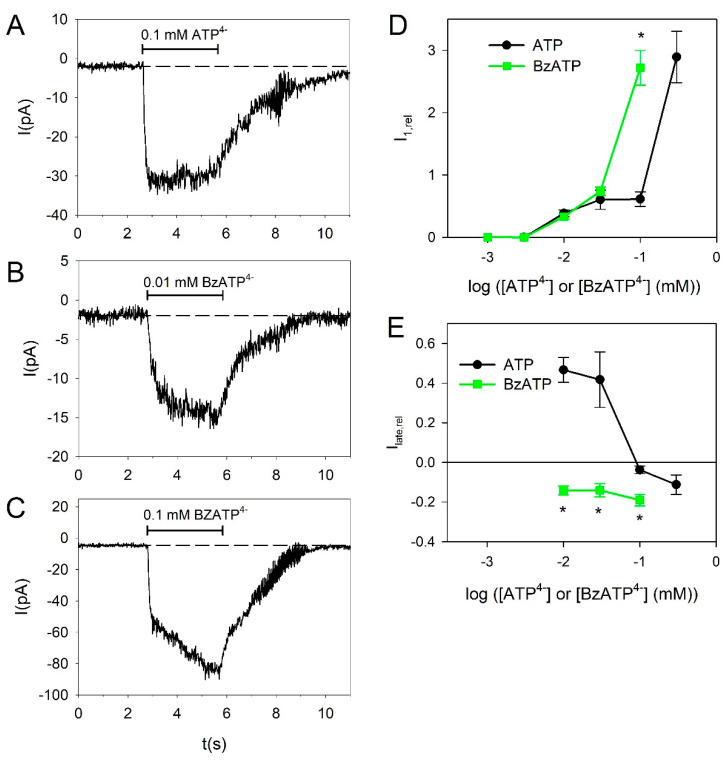
2′(3′)-*O*-(4-Benzoylbenzoyl)adenosine 5′-triphosphate (BzATP)-induced currents in BV-2 microglia cells. (**A**–**C**) Representative current traces induced by application of 0.1 mM ATP^4−^ (**A**), 0.01 mM BzATP^4−^ (**B**) and 0.1 mM BzATP^4−^ (**C**). (**D**,**E**) Comparison of ATP^4−^- and BzATP^4−^-induced current amplitudes (**D**) and late currents I_late,rel_ (**E**). Data for ATP^4−^ are from Figure 1. BzATP: Means ± SEM of 14–35 cells. Significant differences between BzATP^4−^ and ATP^4−^ are marked by asterisks.

Next, we tested the effect of the high-affinity P2X7R agonist 2′(3′)-*O*-(4-Benzoylbenzoyl)adenosine 5′-triphosphate (BzATP) in BV-2 cells. BzATP was significantly more effective than ATP at 0.1 mM, indicating that at this concentration mainly P2X7Rs are activated (Figure 4A,C,D). The action of BzATP becomes clearer when considering its effect on the current kinetics. BzATP shifts the slow current kinetics into a slowly activating kinetics, indicating that BzATP mainly activates the P2X7R-dependent current component (Figure 4A–C,E).

**Figure 5:**
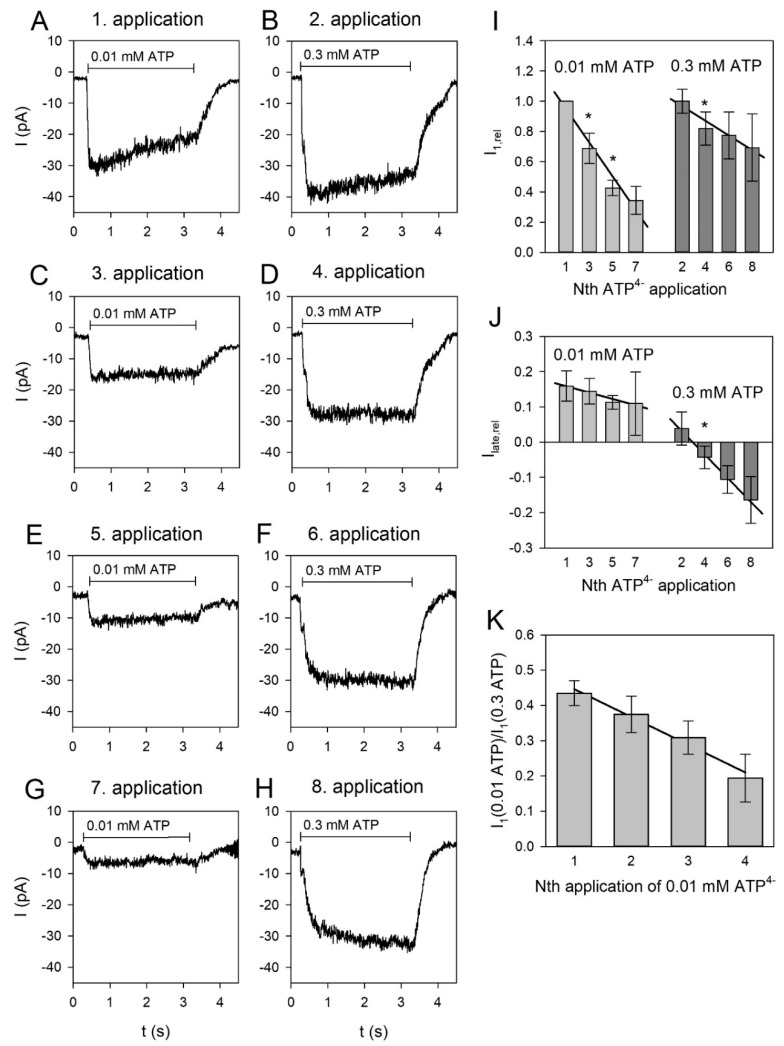
Long-term desensitization of ATP-dependent currents in BV-2 cells. Currents were alternately activated by 0.01 and 0.3 mM ATP^4−^. (**A**–**H**) Representative current traces elicited consecutively by 0.01 and 0.3 mM ATP^4−^ as indicated. The time between the ATP applications was 1 min. (**I**) Dependence of the current amplitudes on the number of ATP applications with significantly different regression coefficients for 0.3 mM ATP^4−^ (−0.11 ± 0.02) and for 0.01 mM ATP^4−^ (−0.25 ± 0.05). The current amplitudes were related to the first application (0.01 mM ATP^4−^). The amplitudes of the currents elicited by 0.3 mM ATP^4−^ were scaled by the amplitude of the current evoked by the first 0.3 mM ATP^4−^ application. (**J**) Application dependence of the relative late currents with the significantly regression coefficients for 0.3 mM ATP^4−^ (−0.07 ± 0.02, significantly different from 0) and for 0.01 ATP^4−^ (−0.019 ± 0.03, not significantly different from 0) (**B**). (**K**) Dependence of the relative current amplitudes I_1_ of the currents elicited by 0.01 mM ATP^4−^ and by the following 0.3 mM ATP^4−^ application. The regression coefficient was −0.072 ± 0.02, which was significantly different from 0. Means ± SEM of 4-312 cells. Significant differences to the previous application are marked by asterisks.

Apart from the short-term kinetics during the 3-s agonist application, the P2X4R can be distinguished from the P2X7R by its long-lasting desensitization behavior [47,48]. Therefore, we repeatedly and alternately applied 0.3 mM and 0.01 mM ATP^4−^. As shown in Figure 5A, currents elicited by 0.01 mM ATP^4−^ showed stronger long-lasting desensitization than currents activated by 0.3 mM ATP^4−^. This agrees with the interpretation that the currents elicited by 0.01 mM ATP^4−^ are to a greater extend mediated by P2X4 receptors than the currents elicited by 0.3 mM ATP^4−^. The late current amplitude I_late,rel_ stayed positive for repeated applications of 0.01 mM ATP^4−^, indicating that despite an accumulation of long-term desensitization, currents evoked by this low [ATP^4−^] are mediated mainly by P2X4 receptors. For currents evoked by applications of 0.3 mM ATP^4−^, I_late,rel_ became progressively negative in repeated applications, indicating a reduction in the P2X4R contribution to the current independent of the P2X7 component (Figure 5B). Accordingly, the relations of the amplitude of the current evoked by 0.01 mM ATP^4−^ to the amplitude of the following current activated by 0.3 mM ATP^4−^ were progressively reduced, with repeated applications indicating a reduction in the P2X4R component independent of the P2X7R component of the current.

### 2.3. Effect of Pro- and Anti-Inflammatory Mediators

**Figure 6:**
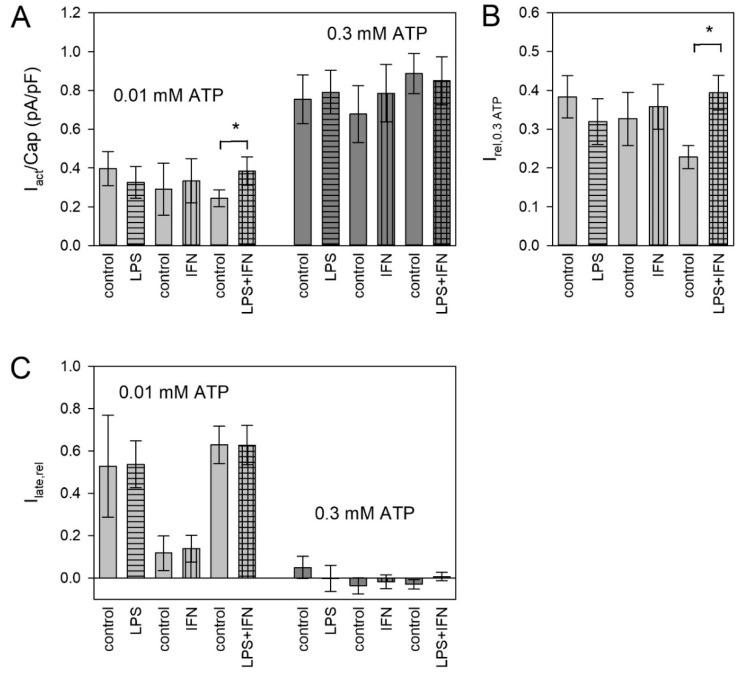
Effect of pro-inflammatory mediators on ATP-induced currents. 10 ng/mL lipopolysaccharide (LPS) and/or interferon gamma (IFN-γ) were applied to the cell culture medium 24 h before the measurements as indicated. (**A**) Current densities measured during application of 0.01 mM or 0.3 mM ATP^4−^ are shown (**B**) Currents evoked by 0.01 mM ATP^4−^ were related to the mean currents evoked by the subsequent application of 0.3 mM ATP^4−^ under the same conditions. (**C**) The relative current amplitudes of the late current I_late,rel_ shown were calculated according to Equation (1). Means ± SEM of 12–24 cells. Significant differences from the control are marked by asterisks.

Mediators involved in inflammation and tissue repair can switch the microglia phenotype between the pro-inflammatory M1 and the anti-inflammatory M2 phenotype [6,49]. We investigated if such mediators could change the functional expression of P2X4 and P2X7 receptors. To induce the M1 phenotype, we used the pro-inflammatory mediators lipopolysaccharide (LPS) and interferon gamma (IFN-γ). Only the combination of LPS and IFN-γ had a significant effect. It increased the amplitude of the current evoked by 0.01 mM ATP^4−^ (Figure 6A) and the relation to the amplitude of the 0.3 mM ATP^4−^ application (Figure 6B). The relative late current was not significantly changed by LPS and IFN-γ (Figure 6C).

**Figure 7:**
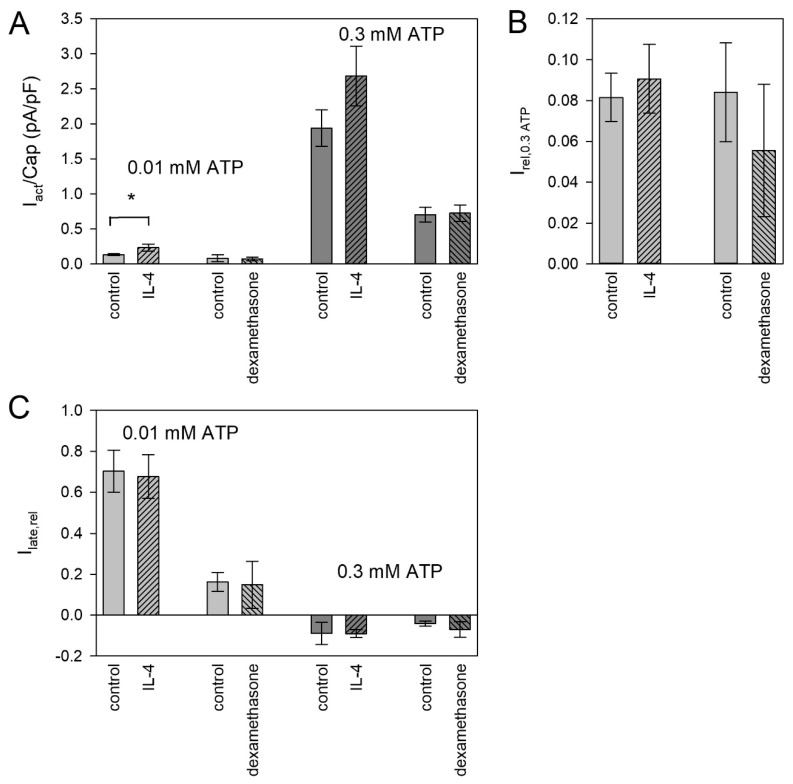
Effect of anti-inflammatory mediators on ATP-induced currents. Shown are (**A**) current densities, (**B**) relative currents and (**C**) relative current amplitudes of the late current I_late,rel_. For further details, see Figure 6. Means ± SEM of 8–16 cells. Significant differences from the control are marked by asterisks.

To induce the M2 phenotype, we used interleukin 4 (IL-4) and dexamethasone. IL-4 increased the currents evoked by 0.01 mM ATP^4−^ (Figure 7A), but not the relation to the subsequent currents activated by 3 mM ATP^4−^ (Figure 7B) and the late current (Figure 7C), indicating that both P2X subtypes are functionally upregulated. Dexamethasone was without effect on both currents, which were elicited by 0.01 and 0.3 mM ATP^4−^.

### 2.4. Effect of Pro- and Anti-Inflammatory Mediators on Volume-Regulated Anion Channels

**Figure 8:**
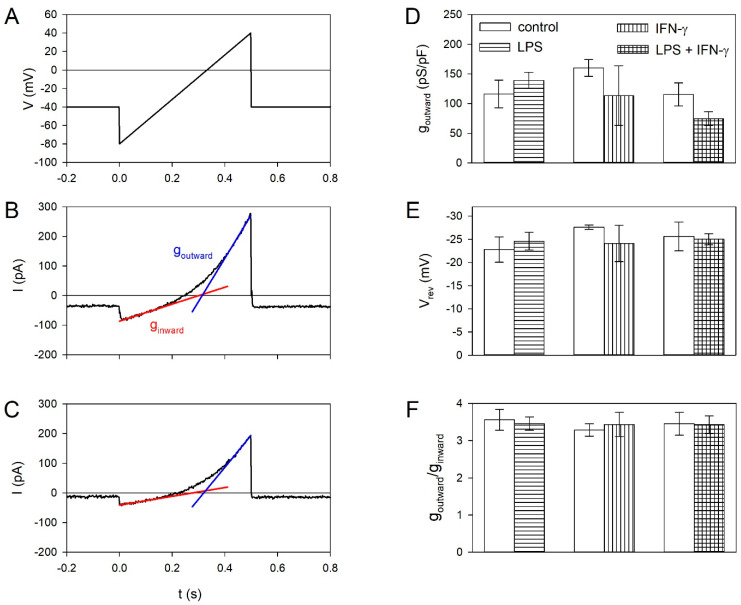
Effect of pro-inflammatory mediators on volume-regulated anion channels (VRACs). (**A**) Ramp protocol. (**B**) Representative ramp currents of control BV-2 cells and (**C**) BV-2 cells following 24 h of incubation with LPS and IFN-γ. The linear approximation to the inward and outward conductance is shown. Dependence of the outward conductance (**D**), the reversal potential V_rev_ (**E**) and the outward rectification (**F**). The assignment of the different column fillings are included in (**D**). Means ± SEM of 6–10 cells.

In macrophages, P2X7Rs require anion channels to promote inflammation [50]. ATP is secreted by macrophages and microglia via volume-regulated anion channels (VRACs) [51,52], which may activate purinergic receptors. We therefore asked whether pro-inflammatory mediators could upregulate VRAC in BV-2 cells to promote purinoceptor-mediated inflammation. However, as shown in Figure 8, LPS and IFN-γ had no significant effect on VRAC-dependent whole cell membrane conductance, VRAC reversal potential and rectification.

## 3. Discussion

### 3.1. ATP-Dependency of Current Amplitudes

The currents in BV-2 cells, in particular the P2X4R component, are small compared with HEK293 cells that express these receptors recombinantly [53]. The current densities also vary considerably depending on the cell batch and the time they are kept in culture (see Figure 7). Therefore, when comparing absolute current densities, e.g., when applying pro- or anti-inflammatory mediators to the cell culture, only cells of the same frozen batch and the same cultivation time following the last cell splitting were compared.

The concentration dependency of the ATP-induced currents clearly shows a biphasic behavior similar to that observed in rat primary microglia cells [54]. A small fraction of the current is evoked with a half maximal effective concentration (EC_50_) of about 10 µM ATP^4−^. This component is at least in part carried by P2X4 receptors, since P2X4 is activated at these low concentrations [14,40,48,55,56,57] and since the P2X4 blocker PSB-15417 [42] significantly reduces its amplitude. A small PSB-15417-unblocked component remains. This could be due to an incomplete block, but this is unlikely since 10 µM PSB-15417 completely blocks human P2X4 receptors [42] and the IC_50_ for ATP-induced Ca^2+^-influx in mP2X4R-expressing HEK cells is <0.1 µM [46]. Another explanation could be an activation of P2X7 receptors via a high affinity activation site reported for the hP2X7R [43]. In mouse peritoneal macrophages, such a component was also observed [58]. At high ATP concentrations, PSB-15417 causes a small, insignificant inhibition of the currents, possibly due to the small contribution of the P2X4R here. To clarify the specificity of PSB-15417, we investigated its effect on the mP2X7R expressed in Xenopus oocytes. The much larger recombinant currents in this preparation decreased the variance of the amplitudes and allowed us to demonstrate a significant blocking effect of PSB-15147 at 10 µM on the mP2X7R.

At ATP concentrations >0.1 mM, there is a drastic increase in the current amplitudes, indicating the activation of the more strongly expressed P2X7R via activation site with low ATP affinity [4,12,54,59,60,61,62]. The existence of functional P2X7 receptors was confirmed here by the activating effect of BzATP, the high affinity P2X7R agonist [12], which was here more effective than 100 µM ATP. However, BzATP was as effective as ATP to evoke currents at 3–30 µM, which may be explained by the nonspecific effect of BzATP, which activates P2X7 but also P2X4 receptors [42,63,64]. Alternatively, BzATP may activate the P2X7R via its high affinity ATP activation site, which has an EC_50_ for ATP similar to the P2X4R [43]. This view is supported by the observation that BzATP did not activate desensitizing (i.e., P2X4R mediated) currents in our preparation.

To block the P2X7R component, the selective P2X7R blocker A438079 was used. A438079 had a strong blocking effect at low ATP concentrations but at >0.3 mM ATP^4−^, the currents that are mainly carried by P2X7 receptors were only partially blocked by A438079. Although A438079 binds to P2X7 receptors at an allosteric binding site [65], indications for a competitive blocking effect were found [66,67]. The partial block can therefore be explained by a competitive displacement of A438079 from its blocking site by ATP.

### 3.2. ATP-Dependency of Current Kinetics

The uncertainties in the pharmacological classification of P2X4R and P2X7R mediated currents prompted us to use other ion current criteria to distinguish P2X4R and P2X7R components. P2X4R-dependent ion currents are characterized by desensitizing kinetics, in contrast to P2X7 receptors, which do not desensitize or even have a slowly activating current component [12,17,19,42,43,45,61,68,69].

At low ATP concentrations (0.01 and 0.03 mM) we measured desensitizing current components that were almost completely blocked by PSB-15147 but not by A438079, indicating that these currents were mainly carried by the P2X4R. Furthermore, at low [ATP^4−^], the slowly activating current component of P2X7R-dependent currents is negligible [43,58] and therefore blocking this component by A438079 has little effect on the current kinetics. At [ATP^4−^] > 0.1 mM, the kinetics switch to slowly activating, indicating that the P2X7R current components predominate. At [ATP^4−^] > 0.03 mM, A438079 shifted the current kinetics towards desensitization, indicating that at these concentrations P2X7 receptors become activated and are blocked by A438079. In addition to its blocking effect on the desensitizing current component at low ATP concentrations, PSB-15147 also appears to block the slowly activating current component at high ATP concentrations (although the effect is not statistically significant, probably due to the large variance of the data at high ATP concentrations.). This is consistent with the partial blocking effect of PSB-15147 on P2X7 receptors. In summary, all pharmacological effects on ATP-induced currents could be explained by distinct effects of the blockers on independent P2X4R and P2X7R current components.

According to the results of the measurements of the concentration–response curves of the ATP-induced current amplitudes and kinetics, we selected 0.01 mM ATP^4−^ to activate mainly P2X4 receptors and 0.3 mM ATP^4−^ to activate mainly P2X7 receptors in BV-2 microglia. To find out whether there is a functional interaction between P2X4 and P2X7 receptors, we investigated whether these current components act in parallel or independently.

### 3.3. Long-Term Desensitization of ATP-Induced Currents

P2X4 receptors show a long-lasting desensitization behavior, which is partly induced by activation-dependent internalization of these receptors with a slow recycling to the cell membrane [31,47,54,57,70,71,72,73]. If the ATP-activated currents were mediated by P2X4/P2X7 heteromers, one would expect only the current amplitudes to decrease, but the kinetics would not change with repeated ATP applications regardless of the ATP concentration. However, currents activated by 0.3 mM ATP^4−^ (carried mainly by P2X7) desensitize more slowly in repeated applications than currents activated by 0.01 mM ATP (carried mainly by P2X4). Additionally, the ratio of the P2X4 current component (activated by 0.01 mM ATP^4−^) to the P2X7 component (activated by 0.3 mM ATP^4−^) is reduced by repeated ATP applications. This indicates that P2X4 receptors desensitize independently of P2X7 receptors, which argues against a significant heteromerization or other physical interaction of P2X4 and P2X7 subunits. Furthermore, the current kinetics change from desensitizing to slowly activating currents with repeated 0.3 mM ATP^4−^ applications. The obvious explanation is that in repeating ATP applications, the P2X4 current component that desensitizes during the ATP application is reduced due to the slow P2X4 recovery from the desensitization independent from the fairly constant P2X7 component. Therefore, the investigation of the slow desensitization kinetics did also not reveal any evidence of functional heteromers between P2X4 and P2X7 subunits with a distinct pharmacologic or kinetic phenotype.

### 3.4. Effect of Pro- and Anti-Inflammatory Mediators on ATP-Induced Currents

Microglia is activated under pathological conditions with changes in the cell phenotype and the expression of P2X4 and P2X7 receptors [3,6,13,14,24,36,49,74]. IFN-γ levels and P2X4 expression in the spinal cord have been shown to be increased following peripheral nerve injury [75]. LPS injection into striatum markedly increased the expression of P2X7R in microglia [76]. Microglial activation and the M1 phenotype can be induced in vivo and in cell cultures by application of LPS and IFN-γ [4,6,14,19,57,77,78,79]. An increase in P2X4 expression in microglia was observed after LPS administration [17,19,80]. However, in alveolar macrophages, IFN-γ and LPS reduced functional and surface area P2X4R expression [57] and in human microglia LPS had no effect on P2X4-dependent ion currents [62]. The functional expression of P2X7 receptors decreased [17,77,79,81], increased [62,76] or was unaltered by LPS [17,19]. The observed effects may be dependent on the LPS concentrations used. We used a rather low concentration (10 ng/mL) to avoid unspecific and toxic effects (see [82]).

In the BV-2 cells investigated here, only the combined application of LPS and IFN-γ had a significant effect, namely a higher number of amoeboid cells with protrusions (see Supplementary Figure S2) and an increase in the expression of functional P2X4 receptors, while the expression of functional P2X7 receptors remained unchanged. This confirms earlier results [17] and indicates an independent expression of the two P2X receptor subtypes. Overall, also these data speak against P2X4/P2X7 heteromers and even against a functional interaction. Previously, we also found no evidence for a functional interaction of co-expressed human P2X1 and P2X7 subunits [83].

The anti-inflammatory IL-4 induces the microglial M2 phenotype [6]. Neuropathic pain is associated with a decreased expression of IL-4 [84], and an intrathecal administration of IL-4 relieved inflammation and hyperalgesia [85]. Therefore, if any, we expected a downregulation of P2X4R or P2X7R by IL-4. Surprisingly, we found the opposite, namely a (statistically significant) increase in the P2X4R-dependent ion current, and a (statistically non-significant) increase in the P2X7R-dependent current. Even though the P2X7R increase was not significant, the unchanged ratio of P2X4R and P2X7R of the ATP-induced currents reinforces the view that the P2X7R is also upregulated.

Activating the microglial glucocorticoid receptor prevented neuronal degeneration triggered by LPS [86]. Accordingly, the anti-inflammatory substance dexamethasone has been reported to decrease the expression of P2X4 receptors in microglia of a rat brain injury model [87]. In our study, dexamethasone had no effect on P2X4 or P2X7 function. This may indicate a principal unresponsiveness of P2X receptor expression to dexamethasone or that most of the BV-2 cells normally exhibit the anti-inflammatory M2 phenotype.

### 3.5. Effect of Pro- and Anti-Inflammatory Mediators on Volume-Regulated Anion Channels

The activation of microglia cells depends on extracellular ATP, which can be released from damaged tissue [3]. Alternatively, microglia cells can themselves release ATP to promote inflammation [4,8,9]. VRAC inhibitors protected the brain from injury in rodent models of transient and permanent cerebral ischemia, implying a major pathological significance of VRACs in stroke [88]. We could show that macrophages [51] and also microglia cells [52] secrete ATP via VRACs. The inflammatory cytokine IFN-γ increased the secretion of ATP from astroglia cells, which then acts on neighboring microglia cells [89]. We therefore asked whether the inflammatory effect of LPS and/or IFN-γ might be mediated by increased VRAC-mediated currents, which was, however, not the case.

## 4. Material and methods

### 4.1. Reagents

All chemicals, except the following, were from Sigma.

DMEM: GIBCO; penicillin, streptomycin: Biochrom; FCS Capricorn Scientific GmbH; Tris: Carl Roth GmbH, Germany; Na-ATP; Roche; EGTA: Serva; A438079 Abcam; interferon γ (IFN-γ): Thermo Fisher Scientific, PSB-15417: Christa Müller (Pharmaceutical Institute, Department of Pharmaceutical & Medicinal Chemistry, University of Bonn, Germany) via Orion (Espoo, Finland).

### 4.2. Cell Culture

Murine microglial BV-2 cells (Banca cellule ICLC, Genova, Italy) were grown at 37 °C in 10% CO_2_ in cell culture medium (DMEM with high glucose) with 4 mM L glutamine, 4.5 g/L glucose, 10% fetal calf serum (FCS), 100 U/mL penicillin, and 100 µg/mL streptomycin. Experiments were performed with cells from 8th to 15th passages.

### 4.3. Synthesis of hP2X7 cRNA

We synthesized capped cRNA as previously described [60] except that we used the anti-reverse cap analog (ARCA, m_2_^7,3′-O^GP_3_G; NU-855; Jena Bioscience, Germany) to ensure the correct cap orientation at the ATG start codon of the cRNA [90,91].

### 4.4. Cloning and Expression of the mP2X7 cDNA

To clone the full-length cDNA encoding the mP2X7 subunit, we isolated total RNA from RAW mouse macrophage cells using the RNeasy kit (Cat. No. 74104, Qiagen, Hilden, Germany). We reverse transcribed RNA into cDNA using the poly d(T)12–18 primers provided with RevertAidTM first strand cDNA synthesis kit (Cat. No. K1611, Fermentas, St. Leon-Rot, Germany). Using mP2X7 gene-specific primers flanked by Gateway AttB1 and AttB2 sequences [92] (Supplementary Table S1), we amplified the mP2X7 cDNA by PCR. We cloned the PCR product directionally with the Gateway cloning system (Invitrogen, Karlsruhe, Germany) into the Gateway pDONR vector, followed by subcloning into pNKS2-GW, a destination vector version of our oocyte expression vector pNKS2 [93]. The mP2X7 cDNA was fully commercially sequenced and differed from the Uniprot sequence Q9Z1M0.2 only in one encoded amino acid residue, P451L. The P451L deviation is known as a natural polymorphism of the mP2X7 gene [94].

### 4.5. Whole Cell Voltage Clamp

Voltage clamp experiments on BV-2 cells were performed in the whole-cell version of the tight-seal patch-clamp technique as described elsewhere [51,58,95]. In short: BV-2 cells, grown on glass coverslips were transferred to a recording chamber, which was perfused with the standard bathing solution (in mM): 140 NaCl, 5.4 KCl, 10 glucose, 10 Hepes, 0.5 MgCl_2_, and 1 CaCl_2_, pH 7.4 adjusted with NaOH (305 mOsmol/kg H_2_O). The patch pipettes were filled with the intracellular solution containing (in mM): 120 Na-aspartate, 10 glucose, 10 Hepes, 3 EGTA, 3 BAPTA, 2 CaCl_2_, 5.5 MgCl_2_, 5 Na-ATP, pH 7.2. The holding potential was −40 mV. For measurements of ATP-dependent currents, the standard bathing solution was exchanged by a low Ca^2+^ bathing solution consisting of (in mM): 140 NaCl, 10 glucose, 10 Hepes, and 0.5 CaCl_2_, pH 7.4. The application of ATP was managed by means of a U-tube [95]. The free concentrations of Ca^2+^ and ATP (ATP^4−^) of the U-tube solution were calculated by a computer program [96]. The P2X4R and P2X7R blockers PSB-15417 and A438079, respectively, were added for 30 s before application of ATP together with the respective blocker to the cell via the U-tube.

The current densities of P2X4- and P2X7-dependent ion currents showed considerable variation. To quantify this, we performed measurements of currents in BV-2 cells induced by 0.01 and 0.3 mM ATP in cells that were kept for different duration in culture. We found that currents induced by both concentrations were increased if the cells were 48 h in culture compared to 24-h culture cells. Additionally, the relation of current amplitudes elicited by 0.01 and 0.3 mM ATP was increased. This is shown in the Supplementary Figure S1. To circumvent a systematic error induced by the different cell batches, the measurement of currents under control conditions and after application of the respective pro- or anti-inflammatory mediator was performed at the same cell batch and in cells which were kept in culture for approximately the same duration. Pro- or antiinflammatory mediators were always applied one day after cell passage and the corresponding measurements were performed at the following day.

For measurements of volume-regulated anion channel (VRAC) currents, the bathing solution was exchanged by an isoosmolar solution (303 mOsmol/kgH_2_O) consisting (in mM) of 140 NaCl, 10 glucose, 10 Hepes, 2 MgCl_2_, and 1 CaCl_2_, pH 7.4. A hypoosmolar solution, also applied via the U-tube was prepared by reducing NaCl to 100 mM (230 mOsmol/kg H_2_O). For measuring cell membrane conductance and current reversal potential, voltage ramps were applied every 1 s going from −80 to +40 mV within 500 ms. For statistical analysis, the whole cell conductance g_outward_ was determined by linear fitting of the current–voltage-relationship in the range of 0 to +40 mV.

The currents were recorded and filtered at 1 kHz using an Axopatch 200A amplifier (Axon instruments, Inc., Foster City, CA, USA) and sampled at 2 kHz. All voltage clamp experiments were performed at room temperature (20–22 °C).

### 4.6. Two-Microelectrode Voltage Clamp

The oocyte treatment and measurements of ion currents dependent on the expression of the murine P2X7 receptor (mP2X7R) in Xenopus oocytes were performed as already described [60]. Female *Xenopus laevis* were kept and ovariectomized as approved by the local animal welfare committee (ref no. 42502-2-1493 MLU) in compliance with EC Directive 86/609/EEC for animal experiments. The oocytes were injected each with 2 ng of the wild-type mP2X7R cRNA and maintained at 19 °C in Barth’s solution (in mM): 100 NaCl, 1 KCl, 1 MgCl_2_, 1 CaCl_2_, pH 7.4) supplemented with penicillin (100 U/mL) and streptomycin (100 µg/mL) until they were used 1–3 days later. Microelectrodes filled with 3 M KCl were impaled into oocytes in frog oocyte Ringer’s solution (ORi, in mM) 90 NaCl, 1 KCl, 1 CaCl_2_, 1 MgCl_2_, 10 HEPES, pH 7.4). The currents were recorded at room temperature using an oocyte clamp OC-725C amplifier (Warner Instruments, Hamden, CT, USA), filtered at 100 Hz and sampled at 85 Hz. Switching between the different bathing solutions was achieved in less than 1 s by a set of computer-controlled magnetic valves using the modified U-tube technique. Measurements of the mP2X7R-dependent currents were performed in bath solutions consisting (in mM) of 100 NaCl, 5 HEPES and 0.1 EGTA, pH 7.4, supplemented with 0.1 mM flufenamic acid to block the conductance caused by the removal of external divalent cations. The mP2X7R-mediated inward currents were elicited by switching for 6 s to a bath solution also containing ATP^4−^ at the concentrations indicated in the text and figures. The interval between agonist applications was usually 3 min.

### 4.7. Data Analysis and Statistics

The data were stored and analyzed on a personal computer. For ion current recording and analysis, a software system developed in our department was used. The SigmaPlot program (SPSS, Chicago, IL, USA) was used for non-linear approximations and graphical representations of the data. The data are given as the mean ± SEM. The statistical data were analyzed using one-way ANOVA. The statistical significance (*p* < 0.05) of the differences between the mean values was tested with the multiple t-test (Bonferroni) of the SigmaPlot program.

## Supplementary Materials

The following are available online at https://www.mdpi.com/1422-0067/21/22/8489/s1.

## Abbreviations

PAMPs	pathogen-associated molecular patterns
DAMPs	danger-associated molecular patterns
P2X4R	P2X4 receptor
mP2X4R	mouse P2X4 receptor
P2X7R	P2X7 receptor
hP2X7R	human P2X7 receptor
LPS	lipopolysaccharide
[ATP^4-^]	free concentration of adenosine triphosphate
BzATP	2′(3′)-*O*-(4-Benzoylbenzoyl)adenosine 5′-triphosphate
IFN-γ	interferon gamma
IL-4	interleukin 4
VRAC	volume-regulated anion channel
EC_50_	half maximal effective concentration
IC_50_	half maximal inhibitory concentration
SEM	standard error of the mean
Hepes	4-(2-hydroxyethyl)-1-piperazineethanesulfonic acid
BAPTA	1,2-bis(o-aminophenoxy)ethane-N,N,N′,N′-tetraacetic acid
FCS	fetal calf serum
EGTA	ethylene glycol-bis(2-aminoethylether)-N N N′N′-tetraacetic acid
DMEM	Dulbecco’s Modified Eagle’s Medium
HEK cells	human embryonic kidney cells
ORi	oocyte Ringer’s solution
ANOVA	analysis of variance
