# Supplementary material for: Dissection of P2X4 and P2X7 Receptor Current Components in BV-2 Microglia
Source: Int J Mol Sci. 2020 Nov 11;21(22):8489. doi: 10.3390/ijms21228489 (PMC7696836; doi:10.3390/ijms21228489)
Supplement: Supplementary file 1 [file ijms-21-08489-s001.pdf]

Supplementary Table S1. Sequences of primers used for RT-PCR amplification and Gateway cloning of mP2X7 DNA

Construct number	Construct name	Primer number	Orienta-tion	Primer sequence
K-4219	mP2X7-pDONR	O-5627	forward	5’ **GGGGACAAGTTTGTACAAAAAAGCAGGCT**ATGCCGGCTTGCTGCAGCTGGAACG
K-4219	mP2X7-pDONR	O-5628	reverse	5’ **GGGGACCACTTTGTACAAGAAAGCTGGGT**TCAGTAGGGATACTTGAAGCCACTG

Construct and primer numbers refer to lab-internal documentation lists and are displayed for traceability. Gateway AttB1 and AttB2 sequences used for cloning into the pDONR vector are shown in bold and underlined. Start and the stop codons are highlighted in green and red in forward and reverse direction.

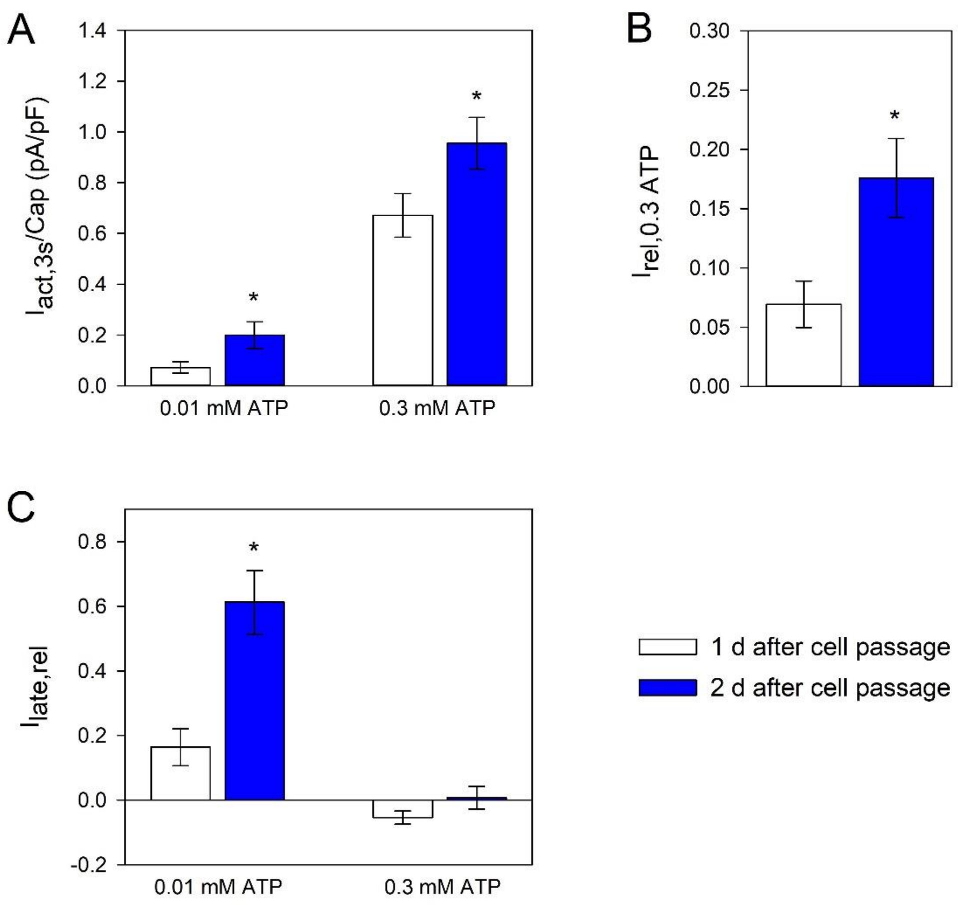

Supplementary Figure S1. Influence of cultivation duration on ATP-induced currents. Shown are (A) current densities of currents activated by the indicated ATP^{4-} concentrations, (B) relative currents I(0.01 ATP)/I(0.3 ATP) and (C) relative current amplitudes of the late current, $I_{late,rel}$. For further details, see Fig. 6. The data a mean values ± SEM of 31-66 cells.

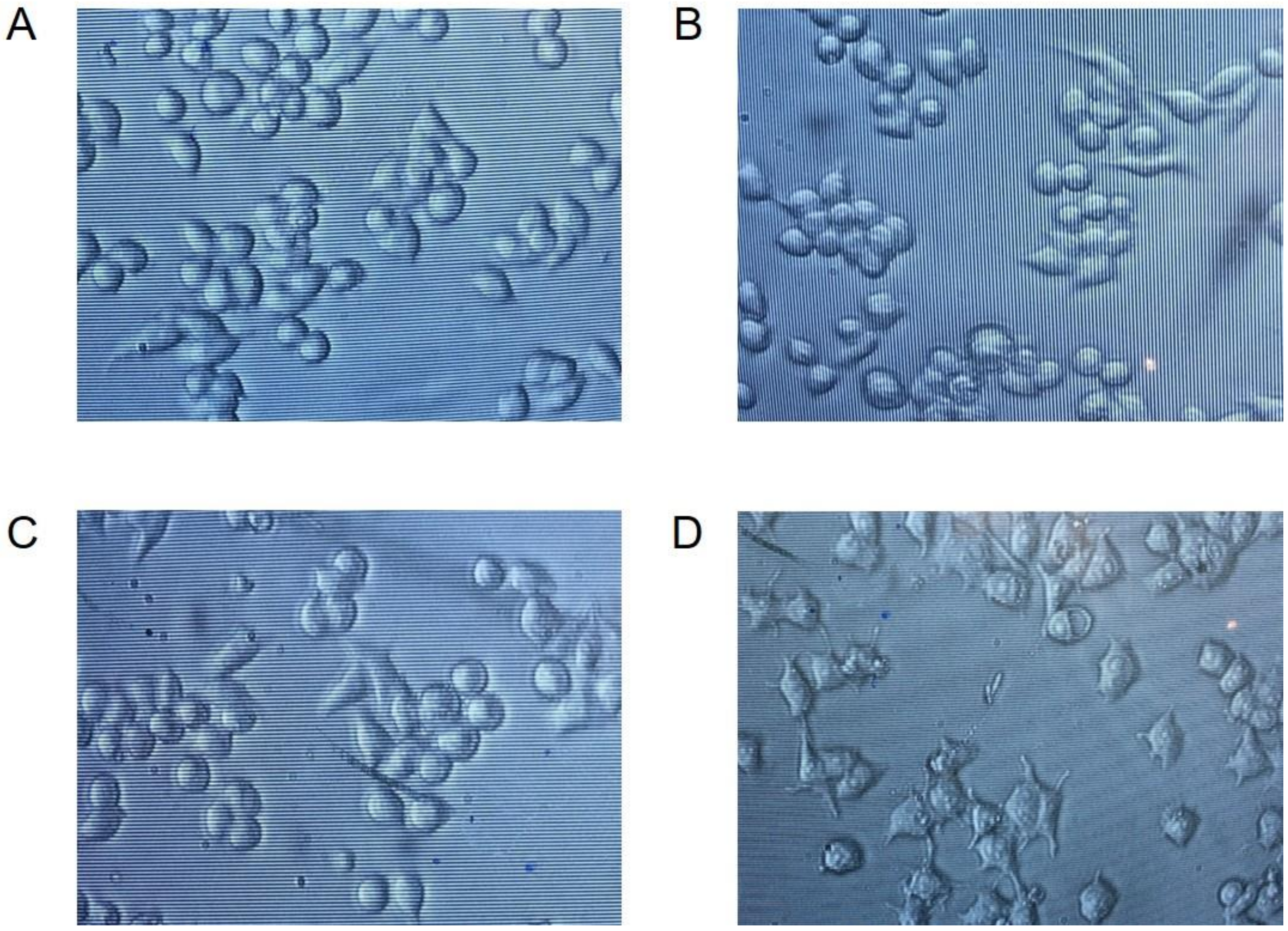

Supplementary Figure S2. Influence of LPS and IFN-γ on cell morphology. Photographs of cell cultures under control condition (A) and after 24 h application of LPS (B), IFN-γ (C) or LPS and IFN-γ (D).
